# Supplementary material for: Differential Metabolomics Reveals Pathogenesis of Pestalotiopsis kenyana Causing Leaf Spot Disease of Zanthoxylum schinifolium
Source: J Fungi (Basel). 2022 Nov 15;8(11):1208. doi: 10.3390/jof8111208 (PMC9698000; doi:10.3390/jof8111208)
Supplement: Supplementary file 1 [file jof-08-01208-s001.zip › Table S1. Common and unique differential metabolites in nine groups..pdf]

Table S1-1 Common and unique differential metabolites in nine groups (positive ion mode)

a.Common metabolites of three groups					
**Description**	**FC**	**Description**	**FC**	**Description**	**FC**
PKP3 vs PKZ3		PKP6 vs PKZ6		PKP15 vs PKZ15	
(2S)-4-Oxo-2-phenyl-3,4-dihydro-2H-chromen-7-yl beta-D-glucopyranoside	0.3554	(2S)-4-Oxo-2-phenyl-3,4-dihydro-2H-chromen-7-yl beta-D-glucopyranoside	0.0261	(2S)-4-Oxo-2-phenyl-3,4-dihydro-2H-chromen-7-yl beta-D-glucopyranoside	0.0247
O-Desmethylvenlafaxine	0.4104	O-Desmethylvenlafaxine	0.0234	O-Desmethylvenlafaxine	0.1669
Estrone	0.3783	Estrone	0.0967	Estrone	0.0273
4-oxo-5-phenylpentanoic acid	0.4179	4-oxo-5-phenylpentanoic acid	0.1688	4-oxo-5-phenylpentanoic acid	0.1276
S-(5-Adenosy)-L-Homocysteine	0.3491	S-(5-Adenosy)-L-Homocysteine	0.0430	S-(5-Adenosy)-L-Homocysteine	0.0296
4-[(4-phenethyl-1,4-diazepan-1-yl)sulfonyl]-2,1,3-benzoxadiazole	0.3724	4-[(4-phenethyl-1,4-diazepan-1-yl)sulfonyl]-2,1,3-benzoxadiazole	0.0282	4-[(4-phenethyl-1,4-diazepan-1-yl)sulfonyl]-2,1,3-benzoxadiazole	0.0755
YNH	0.3570	YNH	0.1992	YNH	0.2837
3-(4-hydroxy-3-methoxyphenyl)propanoic acid	0.2818	3-(4-hydroxy-3-methoxyphenyl)propanoic acid	0.0473	3-(4-hydroxy-3-methoxyphenyl)propanoic acid	0.2104

5-[(Benzoyloxy)methyl]-4,5,6-trihydroxy-2-cyclohexen-1-yl benzoate	0.4210	5-[(Benzoyloxy)methyl]-4,5,6-trihydroxy-2-cyclohexen-1-yl benzoate	0.1487	5-[(Benzoyloxy)methyl]-4,5,6-trihydroxy-2-cyclohexen-1-yl benzoate	0.0677
2-phenyl-4H-furo[2,3-h]chromen-4-one	6.6762	2-phenyl-4H-furo[2,3-h]chromen-4-one	6.2889	2-phenyl-4H-furo[2,3-h]chromen-4-one	3.4339
1-methyl-3,5-di(1-naphthylmethylidene)piperidin-4-one	0.4821	1-methyl-3,5-di(1-naphthylmethylidene)piperidin-4-one	2.7019	1-methyl-3,5-di(1-naphthylmethylidene)piperidin-4-one	3.4666
Scopolamine HBr trihydrate	0.4772	Scopolamine HBr trihydrate	3.2040	Scopolamine HBr trihydrate	0.4589
Sulforidazine	0.5178	Sulforidazine	3.6311	Sulforidazine	3.5567
(5-L-Glutamyl)-L-Amino Acid	2.8190	(5-L-Glutamyl)-L-Amino Acid	2.6944	(5-L-Glutamyl)-L-Amino Acid	3.8875
2-(2-acetyl-3,5-dihydroxyphenyl)acetic acid	0.4127	2-(2-acetyl-3,5-dihydroxyphenyl)acetic acid	0.4823	2-(2-acetyl-3,5-dihydroxyphenyl)acetic acid	0.3825
2-[(carboxymethyl)(methyl)amino]-5-methoxybenzoic acid	0.3242	2-[(carboxymethyl)(methyl)amino]-5-methoxybenzoic acid	2.8119	2-[(carboxymethyl)(methyl)amino]-5-methoxybenzoic acid	3.0941
N-Acetylhistidine	2.5609	N-Acetylhistidine	2.3594	N-Acetylhistidine	4.2823

2-(1H-indol-3-yl)acetic acid	0.4299	2-(1H-indol-3-yl)acetic acid	1.7498	2-(1H-indol-3-yl)acetic acid	1.8358
4-oxo-4-(5,6,7,8-tetrahydronaphthalen-1-ylamino)but-2-enoic acid	2.5986	4-oxo-4-(5,6,7,8-tetrahydronaphthalen-1-ylamino)but-2-enoic acid	2.3740	4-oxo-4-(5,6,7,8-tetrahydronaphthalen-1-ylamino)but-2-enoic acid	5.2933
PS (4:0/16:3)	0.5224	PS (4:0/16:3)	1.7906	PS (4:0/16:3)	1.7669
Traumatic acid	0.5362	Traumatic acid	1.6390	Traumatic acid	2.3397
Lysops 22:5	0.3132	Lysops 22:5	2.0085	Lysops 22:5	3.4228

b. Unique differential metabolites to both groups

**Description**	**FC**	**Description**	**FC**	**Description**	**FC**
PKP3 vs PKZ3		PKP6 vs PKZ6		PKP15 vs PKZ15	
HPK	42.5220	3-(3,4-dimethoxyphenyl)-1-(2-hydroxy-4,6-dimethoxyphenyl)propan-1-one	26.3724	2'-O-Methyladenosine	106.0934
Cytosine	34.1296	Cyclocytidine	25.2060	4-Hydroxytamoxifen	82.0197
5α-Tetrahydrocortisol	33.2998	LLK	24.9814	LDGTS 17:2	70.3735
NMK	31.3431	α-Zearalanol	22.2896	LDGTS 18:1	43.1826
Arachidonoyl ethanolamide phosphate	23.5329	Norbutorphanol	18.7200	N2,N2-Dimethylguanosine	32.5020

5α-Dihydrotestosterone glucuronide	21.0319	Diacetoxyscirpenol	18.0943	ethyl 4-[(6-methyl-3-pyridazinyl)oxy]benzoate	19.1041
CYM-5442	20.5434	TAG (14:0-16:0-18:2)	15.7055	PPK	16.2635
RNH	14.6257	(+)-Evodiamine	11.9432	TAG (16:0-16:0-18:1)	15.3346
6β-Hydroxy-21-desacetyl deflazacort	14.2660	3,6-bis[(1H-indol-3-yl)methyl]piperazine-2,5-dione	11.5920	Phe-Phe	13.3295
ELK	14.1748	2-[(3S)-1-(1-Methyl-4-piperidinyl)-3-pyrrolidinyl]-1H-benzimidazole	10.9931	KMK	11.9071
DGTS (22:0/18:2)	13.5661	2,3-dinor Thromboxane B2	10.9593	4-Nitrosodiphenylamine	11.1948
D-Panthenol	12.9691	N'-{6-[(5-chloro-3-pyridyl)oxy]-3-pyridyl}-N,N-dimethyliminoformamide	10.8705	Morphine D3	10.6059
6-Keto-prostaglandin f1alpha	12.4943	PC (3:0/16:2)	9.9746	PC (3:0/16:2)	9.0632
ANK	11.3395	LDGTS 18:3	9.4950	LPH	8.4242
AMK	11.2557	FPH	8.8116	RMK	8.2795
PPH	10.9384	N,N'-di[4-(2,6-dimethylmorpholino)phenyl]thiourea	8.5942	Epinastine	8.2677

MNK	10.8892	3-(2-Naphthyl)-D-Alanine	8.0721	N1-(3-pyridyl)-4-[(dipropylamino)sulfonyl]benzamide	6.9518
EPK	10.8166	TAG (15:0-18:2-18:2)	7.9324	DLK	6.7466
AKB48 N-(4-hydroxypentyl) metabolite	9.9992	APK	7.7768	Ecgonine methyl ester	6.1132
UR-144 N-(2-hydroxypentyl) metabolite	9.9882	2-(2-amino-3-methylbutanamido)-3-phenylpropanoic acid	7.7087	2-(14,15-Epoxyeicosatrienoyl) glycerol	5.8118

a.Common metabolites of three groups

Description	FC	Description	FC	Description	FC
PKP3 vs PKP6		PKP3 vs PKP15		PKP6 vs PKP15	
-(-)-alpha-Amino-epsilon-Caprolactam	5.8650	L-(-)-alpha-Amino-epsilon-Caprolactam	16.9124	L-(-)-alpha-Amino-epsilon-Caprolactam	2.8836
2-{[methyl(2,3,4,5,6-pentahydroxyhexyl)amino]methylidene}malononitrile	4.5762	2-{[methyl(2,3,4,5,6-pentahydroxyhexyl)amino]methylidene}malononitrile	17.0282	2-{[methyl(2,3,4,5,6-pentahydroxyhexyl)amino]methylidene}malononitrile	3.7210
4-(4-methoxyphenyl)-6-pyridin-4-yl-1,3,5-	0.2498	4-(4-methoxyphenyl)-6-pyridin-4-yl-1,3,5-	0.0618	4-(4-methoxyphenyl)-6-pyridin-4-yl-	0.2475

triazin-2(3H)-one		triazin-2(3H)-one		1,3,5-triazin-2(3H)-one	
mesityl (4-methylphenyl) sulfone	3.5726	mesityl (4-methylphenyl) sulfone	11.2075	mesityl (4-methylphenyl) sulfone	3.1371
Oleanolic acid	2.2223	Oleanolic acid	4.7635	Oleanolic acid	2.1434
L-Pyroglutamic acid	2.3339	L-Pyroglutamic acid	5.3126	L-Pyroglutamic acid	2.2762
3-(dimethylamino)-1-(5-methyl-3-phenyl-4-isoxazolyl)-2-propen-1-one	2.4155	3-(dimethylamino)-1-(5-methyl-3-phenyl-4-isoxazolyl)-2-propen-1-one	5.6490	3-(dimethylamino)-1-(5-methyl-3-phenyl-4-isoxazolyl)-2-propen-1-one	2.3386
N-Acetylsphingosine	0.5891	N-Acetylsphingosine	0.3010	N-Acetylsphingosine	0.5111
Anserine	3.4557	Anserine	9.3706	Anserine	2.7116
Taurolithocholic acid sodium salt	2.7700	Taurolithocholic acid sodium salt	5.7488	Taurolithocholic acid sodium salt	2.0754
Palmitic Acid	0.4003	Palmitic Acid	0.1063	Palmitic Acid	0.2656
3-(3-methylbut-2-en-1-yl)-3H-purin-6-amine	0.3538	3-(3-methylbut-2-en-1-yl)-3H-purin-6-amine	0.0999	3-(3-methylbut-2-en-1-yl)-3H-purin-6-amine	0.2823
N-(4-methoxy-1-methyl-1H-indazol-3-yl)-5-methyl-3-isoxazolecarboxamide	0.1968	N-(4-methoxy-1-methyl-1H-indazol-3-yl)-5-methyl-3-isoxazolecarboxamide	0.4869	N-(4-methoxy-1-methyl-1H-indazol-3-yl)-5-methyl-3-isoxazolecarboxamide	2.4735

b. Unique differential metabolites to both groups

Description	FC	Description	FC	Description	FC

PKP3 vs PKP6		PKP3 vs PKP15		PKP6 vs PKP15	
MPK	109.1210	Laudanosine	372.8409	3,3-dimethyl-5-oxo-5-(2-piperidinoanilino)pentanoic acid	231.7395
Vitexin	46.9546	13,14-dihydro-15-keto-PGD2	183.3651	Solanine	58.7957
Morphine	36.3701	5-Methyl-8-nitro-3-spirocyclohexyl-2,3,4,5-tetrahydro-1H-2-benzazepine	158.3627	13,14-dihydro-15-keto-PGD2	51.9484
CYM-5442	27.2316	Solanine	149.4759	Solanidine	46.0932
PE (18:2/18:3)	23.8710	Solanidine	127.0107	5-(tert-butyl)-N-[4-(trifluoromethyl)phenyl]thiophene-2-carboxamide	34.0241
Arachidonoyl ethanolamide phosphate	21.3515	NMK	89.5911	Orsellinic acid ethyl ester	32.1341
Cytosine	19.7906	5-(tert-butyl)-N-[4-(trifluoromethyl)phenyl]thiophene-2-carboxamide	60.7215	5-chloro-2,8-dimethyl-4-[(3-nitro-2-pyridyl)oxy]quinoline	26.1835
4-Methyl-α-ethyltryptamine	18.1591	CYM-5442	51.4390	Tanespimycin	21.9691
5α-Dihydrotestosterone glucuronide	17.4779	PLK	49.1271	4-[4-chloro-2-nitro-5-(1H-pyrrol-1-	21.1901

				yl)phenyl]morpholine	
NMK	17.0243	Vitexin	49.0782	N1-(1H-1,2,4-triazol-3-yl)-2-hydroxybenzamide	21.0589
5α-Tetrahydrocortisol	16.3831	5α-Tetrahydrocortisol	48.3739	Veratramine	17.2848
PC (18:2e/15:0)	15.4652	4-[(4-phenethyl-1,4-diazepan-1-yl)sulfonyl]-2,1,3-benzoxadiazole	48.3145	2-Acetylpyridine	16.4635
6β-Hydroxy-21-desacetyl deflazacort	15.2840	Morphine	36.7121	Norbutorphanol	16.2785
DAG (15:0/18:2)	14.5549	RMH	36.1905	Flavin Adenine Dinucleotide	16.1963
2-(2,6-dimethoxyphenyl)-5,6-dimethoxy-4H-chromen-4-one	13.7549	Oxohongdenafil	28.9541	Folinic acid	16.0666
ANK	13.0772	DGTS (22:0/18:2)	24.1956	4-(3,4-dihydro-2H-1,5-benzodioxepin-7-ylamino)-4-oxobutanoic acid	12.5485
AMK	12.2692	EKK	23.5680	(3S,9aS)-3-benzyl-octahydro-1H-pyrido[1,2-a]pyrazin-1-one	11.5776
6-Keto-prostaglandin f1alpha	12.2365	S-(5-Adenosy)-L-Homocysteine	22.1183	Emetine	11.2995
4-methyl-5-oxo-2-pentyl-2,5-dihydrofuran-3-	10.1796	5α-Dihydrotestosterone glucuronide	21.3255	UR144 N-(5-hydroxypentyl) metabolite	11.2598

carboxylic acid					
AKB48 N-(4-hydroxypentyl) metabolite	9.4623	Sinomenine	20.1785	3,6-bis[(1H-indol-3-yl)methyl]piperazine-2,5-dione	10.7685

a.Common metabolites of three groups					
**Description**	**FC**	**Description**	**FC**	**Description**	**FC**
PKZ3 vs PKZ6		PKPZ3 vs PKZ15		PKZ6 vs PKZ15	
N-acetyl-L-ornithine	1.9835	N-acetyl-L-ornithine	10.8300	N-acetyl-L-ornithine	5.4601
LDGTS 17:2	3.1748	LDGTS 17:2	103.0692	LDGTS 17:2	32.4650
SPH	0.4534	SPH	9.6294	SPH	7.4315
3-Hydroxypicolinic acid	1.8268	3-Hydroxypicolinic acid	5.5016	3-Hydroxypicolinic acid	3.0116
LDGTS 18:1	16.8308	LDGTS 18:1	475.5962	LDGTS 18:1	28.2575
Sinomenine	2.8726	Sinomenine	21.0530	Sinomenine	7.3290
6-Acetylmorphine	6.7303	6-Acetylmorphine	21.9836	6-Acetylmorphine	3.2663
β-Asarone	0.4513	β-Asarone	0.1111	β-Asarone	0.2462
4-(2,3-dihydro-1,4-benzodioxin-6-	0.3500	4-(2,3-dihydro-1,4-benzodioxin-6-	0.0968	4-(2,3-dihydro-1,4-benzodioxin-6-	0.2764

yl)butanoic acid		yl)butanoic acid		yl)butanoic acid	
ethyl 2-amino-4-(2-thienyl)thiophene-3-carboxylate	4.6562	ethyl 2-amino-4-(2-thienyl)thiophene-3-carboxylate	38.5094	ethyl 2-amino-4-(2-thienyl)thiophene-3-carboxylate	8.2706
6α-Naltrexol	3.0933	6α-Naltrexol	11.5909	6α-Naltrexol	3.7472
DQH	3.5887	DQH	15.9283	DQH	4.4384
Ureidosuccinic acid	1.7354	Ureidosuccinic acid	3.2689	Ureidosuccinic acid	1.8837
L-threo-3-Phenylserine	1.6274	L-threo-3-Phenylserine	3.3724	L-threo-3-Phenylserine	2.0723
PC (3:0/16:2)	8.2977	PC (3:0/16:2)	51.9913	PC (3:0/16:2)	6.2657
methyl 2,5-dimethyl-1H-pyrrole-3-carboxylate	1.6985	methyl 2,5-dimethyl-1H-pyrrole-3-carboxylate	2.6794	methyl 2,5-dimethyl-1H-pyrrole-3-carboxylate	1.5775
4,7-dimethylpyrazolo[5,1-c][1,2,4]triazine-3-carbonitrile	1.6402	4,7-dimethylpyrazolo[5,1-c][1,2,4]triazine-3-carbonitrile	2.5292	4,7-dimethylpyrazolo[5,1-c][1,2,4]triazine-3-carbonitrile	1.5420
3-amino-4-(propylamino)cyclobut-3-ene-1,2-dione	2.0457	3-amino-4-(propylamino)cyclobut-3-ene-1,2-dione	5.0889	3-amino-4-(propylamino)cyclobut-3-ene-1,2-dione	2.4875
4-methoxy-6-(prop-2-en-1-yl)-2H-1,3-benzodioxole	0.3486	4-methoxy-6-(prop-2-en-1-yl)-2H-1,3-benzodioxole	0.1541	4-methoxy-6-(prop-2-en-1-yl)-2H-1,3-benzodioxole	0.4420

methyl 3,4,5-trihydroxycyclohex-1-ene-1-carboxylate	0.5423	methyl 3,4,5-trihydroxycyclohex-1-ene-1-carboxylate	0.2983	methyl 3,4,5-trihydroxycyclohex-1-ene-1-carboxylate	0.5502
2,3-dihydroxypropyl 12-methyltridecanoate	1.6174	2,3-dihydroxypropyl 12-methyltridecanoate	2.7786	2,3-dihydroxypropyl 12-methyltridecanoate	1.7179
N1-(1H-indol-4-yl)cyclohexane-1-carboxamide	2.1259	N1-(1H-indol-4-yl)cyclohexane-1-carboxamide	4.7208	N1-(1H-indol-4-yl)cyclohexane-1-carboxamide	2.2205
5-S-cysteinyldopa	2.0368	5-S-cysteinyldopa	5.4104	5-S-cysteinyldopa	2.6563
Normeperidine	1.8581	Normeperidine	3.9017	Normeperidine	2.0998
(2S)-2-(2-hydroxypropan-2-yl)-2H,3H,7H-furo[3,2-g]chromen-7-one	0.3491	(2S)-2-(2-hydroxypropan-2-yl)-2H,3H,7H-furo[3,2-g]chromen-7-one	0.0739	(2S)-2-(2-hydroxypropan-2-yl)-2H,3H,7H-furo[3,2-g]chromen-7-one	0.2117
5α-Dihydrotestosterone	5.1361	5α-Dihydrotestosterone	17.4101	5α-Dihydrotestosterone	3.3898
cis-4-Hydroxy-D-proline	1.8531	cis-4-Hydroxy-D-proline	4.6355	cis-4-Hydroxy-D-proline	2.5015
DL-Tryptophan	0.2123	DL-Tryptophan	0.0530	DL-Tryptophan	0.2497
Traumatic acid	1.5896	Traumatic acid	3.6029	Traumatic acid	2.2665
2,6-Dihydroxybenzoic acid	1.8608	2,6-Dihydroxybenzoic acid	3.1325	2,6-Dihydroxybenzoic acid	1.6834

Glaucine	2.3384	Glaucine	7.2770	Glaucine	3.1119
Leucine-enkephalin	1.5235	Leucine-enkephalin	3.5507	Leucine-enkephalin	2.3306
Pregnenolone	2.3033	Pregnenolone	5.9435	Pregnenolone	2.5804
Jasmonic acid	0.6026	Jasmonic acid	0.2804	Jasmonic acid	0.4653
1-(2-chlorophenyl)-6,7-dimethoxy-1,4-dihydroisoquinolin-3(2H)-one	0.1911	1-(2-chlorophenyl)-6,7-dimethoxy-1,4-dihydroisoquinolin-3(2H)-one	4.0087	1-(2-chlorophenyl)-6,7-dimethoxy-1,4-dihydroisoquinolin-3(2H)-one	20.9820
(1E,4E)-1,5-bis(4-methoxyphenyl)penta-1,4-dien-3-one	1.7751	(1E,4E)-1,5-bis(4-methoxyphenyl)penta-1,4-dien-3-one	4.1416	(1E,4E)-1,5-bis(4-methoxyphenyl)penta-1,4-dien-3-one	2.3331
Cyclocytidine	37.9393	Cyclocytidine	8.8710	Cyclocytidine	0.2338
Octyl hydrogen phthalate	0.5614	Octyl hydrogen phthalate	0.2770	Octyl hydrogen phthalate	0.4935
3,4,5-Trimethoxyphenyl β-D-glucopyranoside	0.4055	3,4,5-Trimethoxyphenyl β-D-glucopyranoside	0.0607	3,4,5-Trimethoxyphenyl β-D-glucopyranoside	0.1497

b. Unique differential metabolites to both groups

**Description**	**FC**	**Description**	**FC**	**Description**	**FC**
PKZ3 vs PKZ6		PKPZ3 vs PKZ15		PKZ6 vs PKZ15	

LDGTS 16:0	26.3936	LDGTS 17:1	140.8284	Vitexin	100.1629
LDGTS 17:1	21.5017	LDGTS 16:0	127.2284	Emetine	74.4680
LLK	16.3355	Vitexin	108.6972	13,14-dihydro-15-keto-PGD2	38.6106
YLK	15.0632	13,14-dihydro-15-keto-PGD2	41.5953	N1-(3,4,5-trimethoxyphenyl)-3-(2-chlorophenyl)acrylamide	36.5395
Pantetheine	12.6181	2'-O-Methyladenosine	39.2806	Solanidine	25.1998
LDGTS 18:3	11.1383	Oxohongdenafil	36.3837	Solanine	23.3418
TAG (18:2-18:3-18:3)	10.9461	Laudanosine	36.1097	S-Lactoylglutathione	20.9944
5-Methyl-8-nitro-3-spirocyclohexyl-2,3,4,5-tetrahydro-1H-2-benzazepine	9.6036	Shikonin	34.7888	CTP	19.7332
Diacetoxyscirpenol	9.4983	MPK	34.1119	Tanespimycin	15.0323
Senecionine	7.9284	5-Methyl-8-nitro-3-spirocyclohexyl-2,3,4,5-tetrahydro-1H-2-benzazepine	32.6799	(2E)-N-(4-acetamidobutyl)-3-(4-hydroxy-3-methoxyphenyl)prop-2-enamide	14.1271
Norbutorphanol	7.8360	Pantetheine	32.3356	N-Desmethylvenlafaxine	13.7286
Retrorsine	7.4539	Solanidine	24.4374	PQH	13.2643

Oxohongdenafil	7.3546	Solanine	19.8990	4'-Methoxy-α-ethylaminovalerophenone	13.2113
6 β-Hydroxycortisol	6.8536	S-Lactoylglutathione	19.4518	2'-O-Methyladenosine	12.5095
3-(3,4-dimethoxyphenyl)-1-(2-hydroxy-4,6-dimethoxyphenyl)propan-1-one	6.4459	LDGTS 18:3	18.9986	Acetylcholine	12.1751
Ergosterol peroxide	6.3987	6-Keto-prostaglandin f1alpha	17.7117	PC (4:0/16:3)	11.6409
Diflorasone	6.1251	CTP	13.8665	(11E,15Z)-9,10,13-trihydroxyoctadeca-11,15-dienoic acid	11.0474
3-(1-benzylpiperidin-4-yl)-3H-[1,2,3]triazolo[4,5-b]pyridine	5.7541	LPE 22:4	13.8613	Tropine	10.8560
Glycerophospho-N-palmitoyl ethanolamine	5.5267	Thymopentin	13.4839	4-morpholino-3-nitrobenzene-1-sulfonamide	10.5493
LPE 22:5	5.1811	PC (4:0/16:3)	13.2348	5-methoxy-8,8-dimethyl-2-phenyl-4H,8H-pyrano[2,3-h]chromen-4-one	10.3709

Table S1-2. Common and unique differential metabolites in nine groups (negative ion mode)

a.Common metabolites of three groups					
**Description**	**FC**	**Description**	**FC**	**Description**	**FC**
PKP3 vs. PKZ3		PKP6 vs. PKZ6		PKP15 vs. PKZ15	
(+/-)11(12)-EET	0.3521	(+/-)11(12)-EET	2.4529	(+/-)11(12)-EET	2.5204
23-Norcholic acid	0.4190	23-Norcholic acid	2.2299	23-Norcholic acid	4.0001

b. **Unique differential metabolites to both groups**					
**Description**	**FC**	**Description**	**FC**	**Description**	**FC**
PKP3 vs. PKZ3		PKP6 vs. PKZ6		PKP15 vs. PKZ15	
Rutarin	62.6512	6,8-di(tert-butyl)-4-oxo-4H-chromene-2-carboxylic acid	61.5001	Xanthosine	157.9254
Hostanox O3	30.7755	Coenzyme Q1	60.4358	3'-Hydroxystanozolol	55.2217
Aminomalonic acid	22.1110	Gibberellin A4	59.1137	Androsterone glucuronide	15.2596
8-Hydroxyguanosine	16.7937	(+/-)-Cannabichromeorcin	52.3742	Azelaic acid	14.5385
Asparagine	16.4157	Zeranol	29.0396	N-Acetyl-L-histidine	12.5233
D-(+)-Tryptophan	11.2461	Benzyl 6-O-beta-D-glucopyranosyl-beta-D-	17.0667	Salsolinol	12.0544

		glucopyranoside			
Sildenafil-d3	9.2968	N1-cyclohexyl-4-(4-fluorobenzyl)-1,4-diazepane-1-carbothioamide	9.6004	Vitamin B2	11.7548
N-Acetyl-L-histidine	9.1932	3'-Hydroxystanozolol	9.5077	N1-(2-pyridyl)-2-(3,5-difluorophenyl)acetamide	10.4949
2'-Deoxyadenosine 5'-monophosphate (dAMP)	8.4852	Inosine-5'-monophosphate (IMP)	8.9297	N1-cyclohexyl-4-(4-fluorobenzyl)-1,4-diazepane-1-carbothioamide	7.4454
N1-(3-cyano-4,6-diphenyl-2-pyridyl)-4-methylbenzamide	8.0963	N1-(3-amino-4-chlorophenyl)-2-[2,4-di(tert-pentyl)phenoxy]acetamide	7.3286	5-Hydroxymethyluracil	7.2306
N-(1-benzothiophen-3-yl)-N'-(1-benzyl-4-piperidinyl)urea	7.8845	Folic acid	6.7546	Docosahexaenoyl Ethanolamide	7.0888
Gly-Tyr-Ala	7.5185	Feruloyl Putrescine	6.5902	Salvinorin B	6.1297
Deoxyadenosine	7.4527	Deoxyadenosine	6.2007	8-iso-15-keto Prostaglandin E2	5.4396
Prostaglandin A1	7.3718	L-Glutathione (reduced)	6.0419	Caffeine	5.2142
N7-Methylguanosine	6.6791	Prostaglandin K1	5.7676	D-(-)-Fructose	5.1705
Thymidine 5'-monophosphate	6.0751	GlcADG (16:0-18:2)	5.4869	3-benzyl-1-butyl-4-hydroxy-1,2-	4.8619

				dihydroquinolin-2-one	
Phenylacetaldehyde	6.0551	Mycophenolic acid	5.3574	Docosahexaenoic Acid	4.5676
N1-(5-oxo-5H-chromeno[2,3-b]pyridin-7-yl)-4-nitrobenzamide	5.8203	Thymidine	5.2554	Cholic acid	4.3308
Benzyl 6-O-beta-D-glucopyranosyl-beta-D-glucopyranoside	5.7219	Narasin	5.0710	(±)11(12)-DiHET	4.0211
2-{1-[2-(4-benzhydrylpiperazino)-2-oxoethyl]cyclopentyl}acetic acid	5.5094	GlcADG (18:2-18:2)	4.9519	2-(1-{2-[(3-furylmethyl)amino]-2-oxoethyl}cyclohexyl)acetic acid	3.9370

a.Common metabolites of three groups

Description	FC	Description	FC	Description	FC
PKP3 vs. PKP6		PKP3 vs. PKP15		PKP6 vs. PKP15	
5,5-Dimethylhydantoin	3.3892	5,5-Dimethylhydantoin	10.5113	5,5-Dimethylhydantoin	3.1014
D-(-)-Glutamine	3.3594	D-(-)-Glutamine	14.6278	D-(-)-Glutamine	4.3543
Dibenzoyl Thiamine	2.9047	Dibenzoyl Thiamine	7.0451	Dibenzoyl Thiamine	2.4254
Verbascose	5.0204	Verbascose	19.8069	Verbascose	3.9453

Maltotetraose	3.2698	Maltotetraose	8.4133	Maltotetraose	2.5730
D-Ala-D-Ala	4.0528	D-Ala-D-Ala	9.6641	D-Ala-D-Ala	2.3845
Tyrosol	0.5153	Tyrosol	0.3287	Tyrosol	0.6378
LPC 18:3	8.6515	LPC 18:3	56.8028	LPC 18:3	6.5656
9-HpOTrE	4.0075	9-HpOTrE	9.9756	9-HpOTrE	2.4893
Thymidine 5'-monophosphate	3.0396	Thymidine 5'-monophosphate	14.6501	Thymidine 5'-monophosphate	4.8197
(±)9-HpODE	0.5824	(±)9-HpODE	0.2821	(±)9-HpODE	0.4843

b. Unique differential metabolites to both groups

**Description**	**FC**	**Description**	**FC**	**Description**	**FC**
PKP3 vs. PKP6		PKP3 vs. PKP15		PKP6 vs. PKP15	
LPA 18:3	91.4161	Rutarin	132.5498	N'',N'''-di[1-(4-nitrophenyl)ethylidene]carbonic dihydrazide	19.4371
Rutarin	64.8773	LPG 18:3	68.0944	Benzyl 6-O-beta-D-glucopyranosyl-beta-D-glucopyranoside	11.9748
Aminomalonic acid	47.5402	LPI 18:3	67.2374	L-Glutathione (reduced)	11.4574

PA (16:0/18:3)	34.9129	LPE 18:3	62.7999	6,7-Dihydroxycoumarin	11.3955
PA (16:2/18:2)	31.6808	Aminomalonic acid	50.5391	Tauro-alpha-Muricholic acid sodium salt	10.5882
LPE 18:3	30.5079	Piceatannol	33.9669	N3-(4-chlorophenyl)-4-(trifluoromethyl)nicotinamide	10.0018
LPG 18:3	30.0650	LPA 18:3	32.1503	2-[2-(2,3,4-trifluorophenyl)hydrazono]malononitrile	8.7258
LPI 18:3	27.7440	PA (16:0/18:3)	23.7362	1-(2,4-dihydroxyphenyl)-2-(3,5-dimethoxyphenyl)propan-1-one	7.1617
Asparagine	24.0636	N1-(5-oxo-5H-chromeno[2,3-b]pyridin-7-yl)-4-nitrobenzamide	23.6193	8,15-Dihete	6.8343
PA (17:1/18:2)	21.9017	Dodecanedioic acid	22.2194	2'-Deoxyadenosine 5'-monophosphate (dAMP)	6.7673
Testosterone sulfate	19.8544	PA (16:2/18:2)	21.9415	Thromboxane B1	6.5049
PA (16:0/18:2)	18.7880	Hostanox O3	20.3208	Guanosine monophosphate (GMP)	6.4999
PA (14:0/18:2)	17.9167	2'-Deoxyadenosine 5'-monophosphate (dAMP)	16.8463	Inosine-5'-monophosphate (IMP)	6.3397
Hostanox O3	15.1036	PG (18:2/18:3)	14.4456	Genistein	5.5782
Piceatannol	14.7609	Benzyl 6-O-beta-D-glucopyranosyl-beta-D-glucopyranoside	14.2826	Folic acid	5.4115
8-Hydroxyguanosine	13.5663	Testosterone sulfate	13.3091	4-phenyl-2-[(3-pyridylamino)methylidene]cyclohexane-	4.2029

				1,3-dione	
PG (18:2/18:3)	13.4672	Lysopc 18:3	13.1846	Deoxyadenosine	4.1244
LPA 17:2	13.4464	Deoxyadenosine	11.2403	Phloretin	4.0891
N7-Methylguanosine	10.0553	1-(2,4-dihydroxyphenyl)-2-(3,5-dimethoxyphenyl)propan-1-one	11.0452	TPP	4.0868
Oleoyl-L-α-lysophosphatidic acid	9.9657	8,15-Dihete	10.9218	(2R)-2,3-Dihydroxypropanoic acid	3.7585

a.Common metabolites of three groups					
**Description**	**FC**	**Description**	**FC**	**Description**	**FC**
PKZ3 vs. PKZ6		PKZ3 vs. PKZ15		PKZ6 vs. PKZ15	
Caffeine	3.5749	Caffeine	13.6481	Caffeine	3.8178
3-Isopropylmalic acid	2.3267	3-Isopropylmalic acid	4.6066	3-Isopropylmalic acid	1.9799
Prolylleucine	1.5168	Prolylleucine	5.1978	Prolylleucine	3.4269
Mevalonic acid	2.0975	Mevalonic acid	4.4241	Mevalonic acid	2.1092
Apigenin	2.4030	Apigenin	8.3076	Apigenin	3.4571

Dulcitol	1.5788	Dulcitol	2.9870	Dulcitol	1.8919
LPS 18:3	4.2980	LPS 18:3	16.8302	LPS 18:3	3.9158
3-(1-cyano-1,2-dihydroisoquinolin-2-yl)-3-oxopropyl propionate	1.8031	3-(1-cyano-1,2-dihydroisoquinolin-2-yl)-3-oxopropyl propionate	3.6752	3-(1-cyano-1,2-dihydroisoquinolin-2-yl)-3-oxopropyl propionate	2.0383
Undecanedioic acid	6.0091	Undecanedioic acid	27.6346	Undecanedioic acid	4.5988
3-[4-(tert-butyl)anilino]-2-(3-thienylcarbonyl)acrylonitrile	2.2217	3-[4-(tert-butyl)anilino]-2-(3-thienylcarbonyl)acrylonitrile	5.1265	3-[4-(tert-butyl)anilino]-2-(3-thienylcarbonyl)acrylonitrile	2.3075
3-Methyl-2-oxobutanoic acid	0.5888	3-Methyl-2-oxobutanoic acid	0.3241	3-Methyl-2-oxobutanoic acid	0.5504
Glycerol 3-phosphate	0.4681	Glycerol 3-phosphate	0.1861	Glycerol 3-phosphate	0.3975

b. Unique differential metabolites to both groups

**Description**	**FC**	**Description**	**FC**	**Description**	**FC**
PKZ3 vs. PKZ6		PKZ3 vs. PKZ15		PKZ6 vs. PKZ15	
Coenzyme Q1	98.6022	LPC 18:3	78.1745	Genipin	28.7049
Gibberellin A4	82.1640	LPC 16:1	44.1092	LPC 16:1	22.0243
(+/-)-Cannabichromeorcin	61.4006	LPI 18:2	37.0379	Salsolinol	19.5604
Zeranol	22.4005	LPC 16:2	26.0551	D-(-)-Quinic acid	14.5743

LPE 18:3	12.8110	LPE 18:3	24.6795	LPC 16:2	14.4672
tetranor-PGFM	10.9494	D-(-)-Quinic acid	23.2815	LPI 18:2	13.8288
Narasin	9.7056	LPI 18:3	22.8135	Gallic acid	13.0458
FAHFA (18:2/10:0)	9.3528	LPC 18:2	22.7273	LPC 18:3	12.7979
GlcADG (16:0-18:2)	8.0818	Lysopc 18:2	22.6856	3-[3-(beta-D-Glucopyranosyloxy)-2-hydroxyphenyl]propanoic acid	12.4788
Piceatannol	7.5927	3'-Dephosphocoenzyme A	22.0850	Lysopc 18:2	11.7017
2-(1-{2-[(3-furylmethyl)amino]-2-oxoethyl}cyclohexyl)acetic acid	6.3912	tetranor-PGFM	21.5348	diethyl 2-[(4-methoxy-2-nitroanilino)methylidene]malonate	10.7363
N-glycyl-L-proline	5.3298	diethyl 2-[(4-methoxy-2-nitroanilino)methylidene]malonate	20.8301	LPC 18:2	10.0167
FAHFA (18:1/10:0)	5.2115	LPI 18:1	18.9526	Cholic acid	9.4143
3'-Hydroxystanozolol	5.1955	GlcADG (16:0-18:2)	18.6546	4-Hydroxyisoleucine	8.8316
Ursolic acid	5.0929	Gallic acid	18.6002	7-Methyladenine	8.3057
Lysopc 18:3	4.8323	LPE 18:2	18.1507	Prostaglandin E2	7.8355
Rutarin	4.7291	LPC 17:2	14.0807	Glycoursodeoxycholic acid	7.5775

N1-(3-amino-4-chlorophenyl)-2-[2,4-di(tert-pentyl)phenoxy]acetamide	4.2728	7-Methyladenine	13.6767	L-Methionine sulfone	7.4558
L-Glutathione (reduced)	3.8963	Lysopc 18:3	12.8850	Corchorifatty acid F	6.6531
LPE 16:1	3.8552	LPG 18:3	11.3536	O1-(4-chlorobenzoyl)-4-nitrobenzene-1-carbohydroximamide	6.6230
