# Supplementary material for: Differential Metabolomics Reveals Pathogenesis of Pestalotiopsis kenyana Causing Leaf Spot Disease of Zanthoxylum schinifolium
Source: J Fungi (Basel). 2022 Nov 15;8(11):1208. doi: 10.3390/jof8111208 (PMC9698000; doi:10.3390/jof8111208)
Supplement: Supplementary file 1 [file jof-08-01208-s001.zip › Table S2. Enrichment analysis of the top 20 metabolic pathways in nine groups.pdf]

Table S2-1 Enrichment analysis of the top 20 metabolic pathways in nine groups (positive ion mode)

**PKP3 vs. PKZ3**			**PKP6 vs. PKZ6**			**PKP15 vs. PKZ15**		
Pathway Name	Pvalue	Count	Pathway Name	Pvalue	Count	Pathway Name	Pvalue	Count
Monobactam biosynthesis	9.08E-03	4	Oxidative phosphorylation	4.17E-02	2	Tyrosine metabolism	3.39E-02	6
Ubiquinone and other terpenoid-quinone biosynthesis	3.49E-02	3	Riboflavin metabolism	4.17E-02	2	Pyrimidine metabolism	6.07E-02	5
Sulfur relay system	4.85E-02	2	Vitamin B6 metabolism	1.03E-01	3	Cysteine and methionine metabolism	9.05E-02	3
Arginine and proline metabolism	1.13E-01	4	Fatty acid biosynthesis	1.09E-01	2	alpha-Linolenic acid metabolism	9.84E-02	2
Biotin metabolism	1.25E-01	2	Biotin metabolism	1.09E-01	2	Glutathione metabolism	1.37E-01	3
Cysteine and methionine metabolism	1.25E-01	3	Fatty acid elongation	2.06E-01	1	Synthesis and degradation of ketone bodies	1.96E-01	1
Pyrimidine metabolism	1.43E-01	5	Fatty acid degradation	2.06E-01	1	beta-Alanine metabolism	2.53E-01	2
Phenylalanine, tyrosine and tryptophan biosynthesis	1.85E-01	3	Cutin, suberine and wax biosynthesis	2.06E-01	1	Vitamin B6 metabolism	3.34E-01	2
Cyanoamino acid metabolism	2.14E-01	2	Starch and sucrose metabolism	2.06E-01	1	Pentose phosphate pathway	3.54E-01	1
Staurosporine biosynthesis	2.22E-01	1	Sesquiterpenoid and triterpenoid biosynthesis	2.06E-01	1	Valine, leucine and isoleucine degradation	3.54E-01	1
Sesquiterpenoid and triterpenoid biosynthesis	2.22E-01	1	Fatty acid metabolism	2.06E-01	1	Propanoate metabolism	3.54E-01	1
Tryptophan metabolism	2.66E-01	4	Aflatoxin biosynthesis	3.71E-01	1	Thiamine metabolism	3.54E-01	1
beta-Alanine metabolism	3.08E-01	2	Thiamine metabolism	3.71E-01	1	Tryptophan metabolism	4.53E-01	3
Pantothenate and CoA biosynthesis	3.08E-01	2	Purine metabolism	4.33E-01	3	Galactose metabolism	4.82E-01	1
Biosynthesis of amino acids	3.30E-01	5	Phenylalanine metabolism	4.33E-01	3	Amino sugar and nucleotide sugar metabolism	4.82E-01	1
Arachidonic acid metabolism	3.79E-01	3	Galactose metabolism	5.02E-01	1	Carbon metabolism	4.82E-01	1

Aminoacyl-tRNA biosynthesis	3.79E-01	3	alpha-Linolenic acid metabolism	5.02E-01	1	Cyanoamino acid metabolism	5.85E-01	1
Valine, leucine and isoleucine degradation	3.96E-01	1	Methane metabolism	5.02E-01	1	Butanoate metabolism	5.85E-01	1
Lysine biosynthesis	3.96E-01	1	Biosynthesis of unsaturated fatty acids	5.02E-01	1	Glycine, serine and threonine metabolism	1.00E+00	1
D-Arginine and D-ornithine metabolism	3.96E-01	1	Carbon metabolism	5.02E-01	1	Phenylalanine metabolism	1.00E+00	2

**PKP3 vs. PKP6**			**PKP3 vs. PKP15**			**PKP6 vs. PKP15**		
Pathway Name	Pvalue	Count	Pathway Name	Pvalue	Count	Pathway Name	Pvalue	Count
Riboflavin metabolism	7.46E-02	2	Fatty acid biosynthesis	1.56E-02	3	Fatty acid biosynthesis	1.37E-02	3
Biotin metabolism	1.84E-01	2	Biosynthesis of unsaturated fatty acids	1.56E-02	3	Aflatoxin biosynthesis	5.83E-02	2
Biosynthesis of unsaturated fatty acids	1.84E-01	2	Galactose metabolism	1.59E-01	2	Riboflavin metabolism	5.83E-02	2
Arachidonic acid metabolism	2.19E-01	4	Vitamin B6 metabolism	1.73E-01	3	Histidine metabolism	1.56E-01	3
Metabolic pathways	2.59E-01	40	Fatty acid elongation	2.54E-01	1	Vitamin B6 metabolism	1.56E-01	3
Fatty acid elongation	2.75E-01	1	Fatty acid degradation	2.54E-01	1	Fatty acid elongation	2.43E-01	1
Fatty acid degradation	2.75E-01	1	Cutin, suberine and wax biosynthesis	2.54E-01	1	Fatty acid degradation	2.43E-01	1
Cutin, suberine and wax biosynthesis	2.75E-01	1	Starch and sucrose metabolism	2.54E-01	1	Cutin, suberine and wax biosynthesis	2.43E-01	1
Staurosporine biosynthesis	2.75E-01	1	Fatty acid metabolism	2.54E-01	1	One carbon pool by folate	2.43E-01	1
Starch and sucrose metabolism	2.75E-01	1	Purine metabolism	2.78E-01	4	Fatty acid metabolism	2.43E-01	1
Fatty acid metabolism	2.75E-01	1	Metabolic pathways	2.79E-01	37	Oxidative phosphorylation	4.29E-01	1
Butanoate metabolism	3.04E-01	2	Pentose and glucuronate interconversions	4.44E-01	1	Valine, leucine and isoleucine degradation	4.29E-01	1
Porphyrin and chlorophyll metabolism	3.04E-01	2	Aflatoxin biosynthesis	4.44E-01	1	Propanoate metabolism	4.29E-01	1
Cysteine and methionine metabolism	3.49E-01	3	Valine, leucine and isoleucine	4.44E-01	1	Galactose metabolism	5.69E-01	1

			biosynthesis					
Pentose and glucuronate interconversions	4.76E-01	1	Carbapenem biosynthesis	4.44E-01	1	Biosynthesis of unsaturated fatty acids	5.69E-01	1
Oxidative phosphorylation	4.76E-01	1	Nitrogen metabolism	4.44E-01	1	beta-Alanine metabolism	5.97E-01	2
Aflatoxin biosynthesis	4.76E-01	1	beta-Alanine metabolism	6.03E-01	2	Biosynthesis of secondary metabolites	6.11E-01	15
Valine, leucine and isoleucine degradation	4.76E-01	1	Alanine, aspartate and glutamate metabolism	6.45E-01	2	Cysteine and methionine metabolism	6.34E-01	2
Carbapenem biosynthesis	4.76E-01	1	Cysteine and methionine metabolism	6.45E-01	2	Lysine degradation	6.34E-01	2
Taurine and hypotaurine metabolism	4.76E-01	1	Lysine degradation	6.45E-01	2	Glycerophospholipid metabolism	1.00E+00	1

**PKZ3 vs. PKZ6**			**PKZ3 vs. PKZ15**			**PKZ6 vs. PKZ15**		
Pathway Name	Pvalue	Count	Pathway Name	Pvalue	Count	Pathway Name	Pvalue	Count
Lysine degradation	7.83E-02	3	Fatty acid biosynthesis	4.94E-02	3	Histidine metabolism	7.72E-02	4
Fatty acid biosynthesis	8.87E-02	2	Methane metabolism	4.94E-02	3	Vitamin B6 metabolism	7.72E-02	4
alpha-Linolenic acid metabolism	8.87E-02	2	beta-Alanine metabolism	6.36E-02	4	Valine, leucine and isoleucine degradation	9.63E-02	2
Biotin metabolism	8.87E-02	2	Pantothenate and CoA biosynthesis	6.36E-02	4	Propanoate metabolism	9.63E-02	2
Purine metabolism	9.06E-02	4	Phenylalanine, tyrosine and tryptophan biosynthesis	1.03E-01	5	beta-Alanine metabolism	1.77E-01	3
Fatty acid elongation	1.85E-01	1	Pyrimidine metabolism	1.31E-01	7	Pantothenate and CoA biosynthesis	1.77E-01	3
Fatty acid degradation	1.85E-01	1	Oxidative phosphorylation	1.36E-01	2	alpha-Linolenic acid metabolism	2.30E-01	2
Cutin, suberine and wax biosynthesis	1.85E-01	1	Valine, leucine and isoleucine degradation	1.36E-01	2	Synthesis and degradation of ketone bodies	3.12E-01	1
Caffeine metabolism	1.85E-01	1	Propanoate metabolism	1.36E-01	2	Staurosporine biosynthesis	3.12E-01	1
Starch and sucrose metabolism	1.85E-01	1	Riboflavin metabolism	1.36E-01	2	Mannose type O-glycan biosynthesis	3.12E-01	1

Fatty acid metabolism	1.85E-01	1	Lysine degradation	1.96E-01	4	Biosynthesis of secondary metabolites	3.58E-01	20
Alanine, aspartate and glutamate metabolism	3.08E-01	2	Fatty acid elongation	3.70E-01	1	Pentose phosphate pathway	5.28E-01	1
Oxidative phosphorylation	3.37E-01	1	Fatty acid degradation	3.70E-01	1	Pentose and glucuronate interconversions	5.28E-01	1
Valine, leucine and isoleucine biosynthesis	3.37E-01	1	Synthesis and degradation of ketone bodies	3.70E-01	1	Oxidative phosphorylation	5.28E-01	1
Carbapenem biosynthesis	3.37E-01	1	Cutin, suberine and wax biosynthesis	3.70E-01	1	Aflatoxin biosynthesis	5.28E-01	1
Thiamine metabolism	3.37E-01	1	Starch and sucrose metabolism	3.70E-01	1	Carbapenem biosynthesis	5.28E-01	1
Riboflavin metabolism	3.37E-01	1	Mannose type O-glycan biosynthesis	3.70E-01	1	Taurine and hypotaurine metabolism	5.28E-01	1
Arginine and proline metabolism	3.71E-01	3	Linoleic acid metabolism	3.70E-01	1	Thiamine metabolism	5.28E-01	1
Galactose metabolism	4.61E-01	1	Fatty acid metabolism	3.70E-01	1	Riboflavin metabolism	5.28E-01	1
C5-Branched dibasic acid metabolism	4.61E-01	1	Phenylalanine metabolism	5.03E-01	5	Nitrogen metabolism	5.28E-01	1

Table S2-2 Enrichment analysis of the top 20 metabolic pathways in nine groups (negative ion mode)

**PKP3 vs. PKZ3**			**PKP6 vs. PKZ6**			**PKP15 vs. PKZ15**		
Pathway Name	Pvalue	Count	Pathway Name	Pvalue	Count	Pathway Name	Pvalue	Count
Lysine degradation	9.08E-02	2	Inositol phosphate metabolism	4.76E-02	2	Biosynthesis of unsaturated fatty acids	9.58E-02	4
Starch and sucrose metabolism	9.08E-02	2	Caffeine metabolism	1.23E-01	2	Caffeine metabolism	1.14E-01	2
Folate biosynthesis	9.08E-02	2	Thiamine metabolism	1.23E-01	2	Arginine and proline metabolism	1.99E-01	2
Amino sugar and nucleotide sugar metabolism	1.61E-01	2	Riboflavin metabolism	1.23E-01	2	Amino sugar and nucleotide sugar metabolism	1.99E-01	2
Phenylalanine metabolism	1.72E-01	3	Glycolysis / Gluconeogenesis	2.12E-01	2	Arachidonic acid metabolism	1.99E-01	2
ABC transporters	1.72E-01	3	Oxidative phosphorylation	2.12E-01	2	Phosphonate and phosphinate metabolism	2.13E-01	1
Fatty acid degradation	1.89E-01	1	Arginine and proline metabolism	2.12E-01	2	Glycerophospholipid metabolism	2.13E-01	1
Biosynthesis of siderophore group nonribosomal peptides	1.89E-01	1	Phosphonate and phosphinate metabolism	2.21E-01	1	Linoleic acid metabolism	2.13E-01	1
Aminoacyl-tRNA biosynthesis	3.16E-01	2	One carbon pool by folate	2.21E-01	1	Taurine and hypotaurine metabolism	2.88E-01	2
2-Oxocarboxylic acid metabolism	3.16E-01	2	RNA transport	2.21E-01	1	Phenylalanine, tyrosine and tryptophan biosynthesis	3.82E-01	1
Ascorbate and aldarate metabolism	3.43E-01	1	Endocytosis	2.21E-01	1	Sphingolipid metabolism	3.82E-01	1
Phenylalanine, tyrosine and tryptophan biosynthesis	3.43E-01	1	Purine metabolism	2.26E-01	4	Diterpenoid biosynthesis	3.82E-01	1
Purine metabolism	3.97E-01	3	Biosynthesis of secondary metabolites	2.73E-01	11	Galactose metabolism	5.16E-01	1
Galactose metabolism	4.69E-01	1	Metabolic pathways	3.19E-01	21	Riboflavin metabolism	5.16E-01	1
Glutathione metabolism	4.69E-01	1	Fructose and mannose metabolism	3.95E-01	1	Sulfur metabolism	5.16E-01	1
Pantothenate and CoA biosynthesis	4.69E-01	1	Valine, leucine and isoleucine	3.95E-01	1	Vancomycin resistance	5.16E-01	1

			biosynthesis					
Biosynthesis of antibiotics	5.62E-01	5	Phenylalanine, tyrosine and tryptophan biosynthesis	3.95E-01	1	ABC transporters	6.78E-01	2
Glycolysis / Gluconeogenesis	5.71E-01	1	Terpenoid backbone biosynthesis	3.95E-01	1	Metabolic pathways	7.00E-01	19
Arginine and proline metabolism	5.71E-01	1	Diterpenoid biosynthesis	3.95E-01	1	Glycolysis / Gluconeogenesis	1.00E+00	1
Tyrosine metabolism	5.71E-01	1	Pyrimidine metabolism	4.13E-01	3	Pyrimidine metabolism	1.00E+00	2

**PKP3 vs. PKP6**			**PKP3 vs. PKP15**			**PKP6 vs. PKP15**		
Pathway Name	Pvalue	Count	Pathway Name	Pvalue	Count	Pathway Name	Pvalue	Count
Vancomycin resistance	1.38E-02	3	Galactose metabolism	2.63E-02	3	Biosynthesis of unsaturated fatty acids	9.91E-03	6
Oxidative phosphorylation	4.56E-02	3	Tyrosine metabolism	8.27E-02	3	Amino sugar and nucleotide sugar metabolism	5.49E-02	3
Tyrosine metabolism	4.56E-02	3	Valine, leucine and isoleucine biosynthesis	9.02E-02	2	Terpenoid backbone biosynthesis	6.72E-02	2
Amino sugar and nucleotide sugar metabolism	4.56E-02	3	Terpenoid backbone biosynthesis	9.02E-02	2	Valine, leucine and isoleucine degradation	1.68E-01	2
Nicotinate and nicotinamide metabolism	6.11E-02	4	Biosynthesis of unsaturated fatty acids	1.28E-01	5	Thiamine metabolism	1.68E-01	2
Histidine metabolism	9.48E-02	3	Purine metabolism	1.69E-01	5	Fatty acid biosynthesis	2.62E-01	1
ABC transporters	1.01E-01	4	Nicotinate and nicotinamide metabolism	1.97E-01	4	Cutin, suberine and wax biosynthesis	2.62E-01	1
Lysine degradation	1.49E-01	2	Glutathione metabolism	2.18E-01	2	One carbon pool by folate	2.62E-01	1
Glutathione metabolism	1.49E-01	2	Starch and sucrose metabolism	2.18E-01	2	Biotin metabolism	2.62E-01	1
Starch and sucrose metabolism	1.49E-01	2	Butanoate metabolism	2.18E-01	2	RNA transport	2.62E-01	1
Butanoate metabolism	1.49E-01	2	Thiamine metabolism	2.18E-01	2	Endocytosis	2.62E-01	1

Aminoacyl-tRNA biosynthesis	1.58E-01	3	Vitamin B6 metabolism	2.18E-01	2	Glycolysis / Gluconeogenesis	2.81E-01	2
Fatty acid degradation	2.46E-01	1	Vancomycin resistance	2.18E-01	2	Pentose phosphate pathway	2.81E-01	2
Ascorbate and aldarate metabolism	4.33E-01	1	Fatty acid biosynthesis	3.03E-01	1	Fructose and mannose metabolism	4.57E-01	1
Arginine biosynthesis	4.33E-01	1	Cutin, suberine and wax biosynthesis	3.03E-01	1	Valine, leucine and isoleucine biosynthesis	4.57E-01	1
Monobactam biosynthesis	4.33E-01	1	Glycerophospholipid metabolism	3.03E-01	1	Lysine biosynthesis	4.57E-01	1
Valine, leucine and isoleucine biosynthesis	4.33E-01	1	RNA transport	3.03E-01	1	Tryptophan metabolism	4.57E-01	1
Cyanoamino acid metabolism	4.33E-01	1	Endocytosis	3.03E-01	1	Inositol phosphate metabolism	4.57E-01	1
Sphingolipid metabolism	4.33E-01	1	Alanine, aspartate and glutamate metabolism	4.32E-01	3	beta-Alanine metabolism	6.06E-01	2
Cysteine and methionine metabolism	5.96E-01	2	Fructose and mannose metabolism	5.16E-01	1	2-Oxocarboxylic acid metabolism	6.52E-01	2

**PKZ3 vs. PKZ6**			**PKZ3 vs. PKZ15**			**PKZ6 vs. PKZ15**		
Pathway Name	Pvalue	Count	Pathway Name	Pvalue	Count	Pathway Name	Pvalue	Count
Biosynthesis of secondary metabolites	6.92E-03	14	Thiamine metabolism	3.35E-02	3	Oxidative phosphorylation	6.71E-03	4
Galactose metabolism	8.81E-03	3	Metabolic pathways	7.63E-02	32	Phenylalanine metabolism	4.82E-02	5
Valine, leucine and isoleucine biosynthesis	4.40E-02	2	Oxidative phosphorylation	1.03E-01	3	Tyrosine metabolism	7.66E-02	3
Lysine biosynthesis	4.40E-02	2	Tyrosine metabolism	1.03E-01	3	Arachidonic acid metabolism	7.66E-02	3
Terpenoid backbone biosynthesis	4.40E-02	2	Valine, leucine and isoleucine biosynthesis	1.06E-01	2	Caffeine metabolism	2.08E-01	2
ABC transporters	6.25E-02	4	Inositol phosphate metabolism	1.06E-01	2	Valine, leucine and isoleucine degradation	2.08E-01	2
2-Oxocarboxylic acid metabolism	1.10E-01	3	Terpenoid backbone biosynthesis	1.06E-01	2	Propanoate metabolism	2.08E-01	2

Caffeine metabolism	1.14E-01	2	Phenylalanine metabolism	1.12E-01	5	Butanoate metabolism	2.08E-01	2
Starch and sucrose metabolism	1.14E-01	2	Galactose metabolism	2.50E-01	2	Thiamine metabolism	2.08E-01	2
Pantothenate and CoA biosynthesis	1.14E-01	2	Valine, leucine and isoleucine degradation	2.50E-01	2	Sulfur metabolism	2.08E-01	2
Metabolic pathways	1.83E-01	21	Butanoate metabolism	2.50E-01	2	Vancomycin resistance	2.08E-01	2
Pentose phosphate pathway	1.99E-01	2	Riboflavin metabolism	2.50E-01	2	Cutin, suberine and wax biosynthesis	2.95E-01	1
Linoleic acid metabolism	2.13E-01	1	Vitamin B6 metabolism	2.50E-01	2	Linoleic acid metabolism	2.95E-01	1
Biotin metabolism	2.13E-01	1	Purine metabolism	2.94E-01	5	Porphyrin and chlorophyll metabolism	2.95E-01	1
RNA transport	2.13E-01	1	Cutin, suberine and wax biosynthesis	3.28E-01	1	Citrate cycle (TCA cycle)	3.59E-01	3
Endocytosis	2.13E-01	1	RNA transport	3.28E-01	1	Pyruvate metabolism	4.20E-01	3
beta-Alanine metabolism	2.88E-01	2	Endocytosis	3.28E-01	1	Nicotinate and nicotinamide metabolism	4.20E-01	3
Fructose and mannose metabolism	3.82E-01	1	2-Oxocarboxylic acid metabolism	3.92E-01	3	Biosynthesis of unsaturated fatty acids	4.47E-01	4
Arginine biosynthesis	3.82E-01	1	Pyrimidine metabolism	4.73E-01	4	Arginine biosynthesis	5.05E-01	1
Monobactam biosynthesis	3.82E-01	1	Fructose and mannose metabolism	5.50E-01	1	Valine, leucine and isoleucine biosynthesis	5.05E-01	1
